# Characterization of CD8^+^ T-Cell Responses in the Peripheral Blood and Skin Injection Sites of Melanoma Patients Treated with mRNA Electroporated Autologous Dendritic Cells (TriMixDC-MEL)

**DOI:** 10.1155/2013/976383

**Published:** 2013-01-03

**Authors:** Daphné Benteyn, An M. T. Van Nuffel, Sofie Wilgenhof, Jurgen Corthals, Carlo Heirman, Bart Neyns, Kris Thielemans, Aude Bonehill

**Affiliations:** ^1^Laboratory of Molecular and Cellular Therapy, Department of Immunology-Physiology and Dendritic Cell Bank, Vrije Universiteit Brussel, 1090 Brussels, Belgium; ^2^Department of Medical Oncology, Universitair Ziekenhuis Brussel, 1090 Brussels, Belgium

## Abstract

Treatment of melanoma patients with mRNA electroporated dendritic cells (TriMixDC-MEL) stimulates T-cell responses against the presented tumor-associated antigens (TAAs). In the current clinical trials, melanoma patients with systemic metastases are treated, requiring priming and/or expansion of preexisting TAA-specific T cells that are able to migrate to both the skin and internal organs. We monitored the presence of TAA-specific CD8^+^ T cells infiltrating the skin at sites of intradermal TriMixDC-MEL injection (SKILs) and within the circulation of melanoma patients treated in two clinical trials. In 10 out of fourteen (71%) patients screened, CD8^+^ T cells recognizing any of the four TAA presented by TriMixDC-MEL cellular vaccine were found in both compartments. In total, 30 TAA-specific T-cell responses were detected among the SKILs and 29 among peripheral blood T cells, of which 24 in common. A detailed characterization of the antigen specificity of CD8^+^ T-cell populations in four patients indicates that the majority of the epitopes detected were only recognized by CD8^+^ T cells derived from either skin biopsies or peripheral blood, indicating that some compartmentalization occurs after TriMix-DC therapy. To conclude, functional TAA-specific CD8^+^ T cells distribute both to the skin and peripheral blood of patients after TriMixDC-MEL therapy.

## 1. Introduction

Several cancer immunotherapeutic approaches are currently under investigation, amongst which dendritic-cell-based immunotherapy. Dendritic cells (DCs) are potent antigen-presenting cells that can easily be loaded with antigens. Recent improvements of DC therapy include the use of mRNA encoding full-length tumor antigen(s) instead of peptides to load DCs for clinical trials. This results in broader T-cell responses [1–3] and avoids the limitation of known peptides and matching HLA phenotypes.

Monitoring TAA-restricted T-cell responses during treatment is of great importance to investigate the immunogenicity of the vaccine and the potential correlation between the immune response and the clinical outcome of the patients and also for future treatment design. Ideally, immune responses should be monitored within the tumor, but this site is not always accessible. Alternative methods are the characterization of circulating treatment-specific CD8^+^  T cells in the peripheral blood [4–6], or the characterization of treatment-specific skin infiltrating lymphocytes (SKILs) at delayed type hypersensitivity (DTH) sites [7, 8]. Both compartments are easily accessible and have advantages and limitations. Immune monitoring of skin biopsies can be performed without prior *in vitro* T-cell restimulation and highlights the migratory potential of the antigen-specific CD8^+^  T cells after treatment, but only a limited amount of cells is available. In contrast, peripheral blood screening requires several *in vitro* restimulations to uncover low frequencies of specific CD8^+^ T cells; however, enough material is available and pretreatment immune monitoring can be performed without additional invasive intervention. Indeed, all patients undergo a leukapheresis before treatment for the generation of the TriMixDC-MEL vaccine. The remainder of the material is then used for further immune monitoring. 

Since, in advanced cancer patients, tumors are located at different anatomical locations, it is of great importance that T cells have the capacity to migrate to and eradicate tumor cells at different tissue sites. In a mouse study, it has been shown by our group [9] that immunization with TriMix mRNA results in antigen-specific CD8^+^ T cells located in different organs, including the lymph nodes, spleen, and peripheral blood, highlighting the capacity of the T cells to migrate to different body sites. 

With this project, we set out to characterize the immune responses in skin biopsies and peripheral blood of melanoma patients treated with TriMixDC-MEL. 

## 2. Materials and Methods

### 2.1. Patients, Vaccine Preparation, and Treatment Schedule

Fourteen patients with recurrent stage III or stage IV melanoma were recruited in two institutional (UZ Brussels) pilot clinical trials on autologous TriMix-DC treatment (EudraCT2009-015748-40/NCT01066390) [10]. TriMix-DCs were manufactured according to a previously described protocol [11]. In brief, immature DCs were coelectroporated with TriMix mRNA (a combination of CD40L, caTLR4, and CD70 encoding mRNA) in combination with one of four mRNAs encoding a TAA (tyrosinase, MAGE-A3, MAGE-C2, or gp100) linked to an HLA class II targeting signal. Genetic constructs encoding these different mRNAs have been described previously [1]. After electroporation, the four different TriMixDC-MEL cellular constituents (i.e., DCs expressing one of the four antigens) were mixed at equal ratios and cryopreserved. Before treatment, an in-process quality control check was performed as well as a quality control check of the final cellular product. The cellular product was thawed 2 to 3 hours before injection. Each patient received 4 DC injections on a biweekly basis, after which immunomonitoring was performed [10]. Patients 72 to 98 (Table 1) received 4 times ±43 × 10^6^ DC intradermally (ID), whereas the next four patients (102–116) received a combination of intradermal and intravenous (IV) DCs, whereas the last patient (125) received intravenous DCs only.

### 2.2. Monitoring Treatment-Specific T-Cell Responses in Peripheral Blood

Immune monitoring of the peripheral blood of the patients was performed as described elsewhere [12]. Pretreatment and posttreatment samples were taken before the first injection and 1 week after the fourth administration, respectively. Briefly, CD8^+^ T cells were isolated from the nonadherent fraction of the patient's leukapheresis product before and after DC treatment using anti-CD8^+^ MACS beads (Miltenyi), obtaining a >90% purity (data not shown).

Twenty million CD8^+^  T cells were cocultured with autologous TriMix-DC coelectroporated with one of 4 different tumor antigens at a 10 : 1 ratio in stimulation medium (IMDM, 1% human AB serum, 1 mM sodium pyruvate, nonessential amino acids, 0.24 mM L-asparagine, and 0.55 mM L-arginine (all from Lonza, Verviers, Belgium)) without any further addition of exogenous cytokines. CD8^+^ T cells were restimulated weekly. The culture medium was changed when necessary, and after 2 and 3 rounds of stimulation CD8^+^ T cells were harvested and their antigen specificity and function were determined. To this end, they were stimulated overnight with autologous Epstein-Barr Virus-transformed B cells (aEBV-B) that were electroporated with the treatment TAAs. Upregulation of CD137, cytokine secretion, and intracellular cytokines were investigated in response to antigen-specific stimulation. To identify the epitopes recognized by the CD8^+^  T cells, overnight restimulation was performed with aEBV-B cells pulsed with pools of 10 peptides (10 *μ*g/mL in stimulation medium), or with the individual peptides composing the recognized pools. Peptides were 15 mers, each with 11 amino acids (aa) overlap, and in total spanning the entire TAA sequence. The gp100 protein was spanned by 163 synthetic peptides (16 pools), tyrosinase by 130 peptides (13 pools), MAGE-C2 by 91 peptides (9 pools), and MAGE-A3 by 76 peptides (8 pools) (all purchased from EMC Microcultures, Tübingen, Germany). Responses were scored based on the percentage CD137 expression and intracellular IFN*γ*/TNF*α* (weak (<10%), moderate (10–20%), or strong (>20%)). Peptides were considered positive, when the cytokine secretion was increased 2.5-fold compared to a control peptide. 

### 2.3. Monitoring Treatment-Specific T-Cell Responses in Skin Biopsies

Immunomonitoring was performed as described elsewhere [8]. Briefly, 1 week after the fourth DC administration, a small amount of TriMixDC-MEL was injected intradermally to induce a delayed type IV hypersensitivity response from which skin biopsies were taken 48 or 72 hours later. After 2.5 weeks of culture in IL-2 (100 IU/mL) supplemented medium, SKILs were harvested and their antigen specificity was determined as for the blood-derived CD8^+^ T cells. 

## 3. Results and Discussion

Immune monitoring of melanoma patients after DC treatment was performed on skin biopsies and on peripheral blood. For 14 patients, we had sufficient material to investigate the presence of antigen-specific CD8^+^ T cells in both compartments. Cytotoxic CD8^+^  T-cells are known as key players in tumor immunity. Upon stimulation by recognition of TAA-derived peptides presented by antigen presenting cells, they are capable to kill tumor cells while sparing normal cells, resulting in minimal tissue damage. It is thus important to characterize the CD8^+^ T-cell immune response after DC immunotherapy in order to gain knowledge about their capacity to recognize and possibly eradicate tumor cells. An overview of the results is given in Table 1. 

Three patients included in this study did not have treatment antigen-specific T cells in the skin biopsies. Accordingly, in 2 of them, no specific T cells could be detected in the peripheral blood. In the third patient (116), although the responses were weak, peripheral CD8^+^ T cells recognizing gp100, MAGE-C2, and tyrosinase were found after DC treatment. In contrast, for patient 76, no treatment-specific T cells were found in the blood, whereas a weak tyrosinase-specific response was found in the skin (Table 1). 

In the 14 patients tested, we detected 30 positive responses directed against the treatment antigens in the skin biopsies and 29 responses in the peripheral blood, of which 24 in common. 

As the patients are treated with an equal ratio mixture of DCs each expressing one out of four different treatment antigens, we also analyzed the responses per antigen. In total, 1/4 gp100-reponders (25%), 8/12 tyrosinase-responders (67%), 9/11 MAGE-C2-responders (82%), and 7/8 MAGE-A3-responders (88%) had CD8^+^  T cells that could be detected or found in both compartments. 

**Figure 1:**
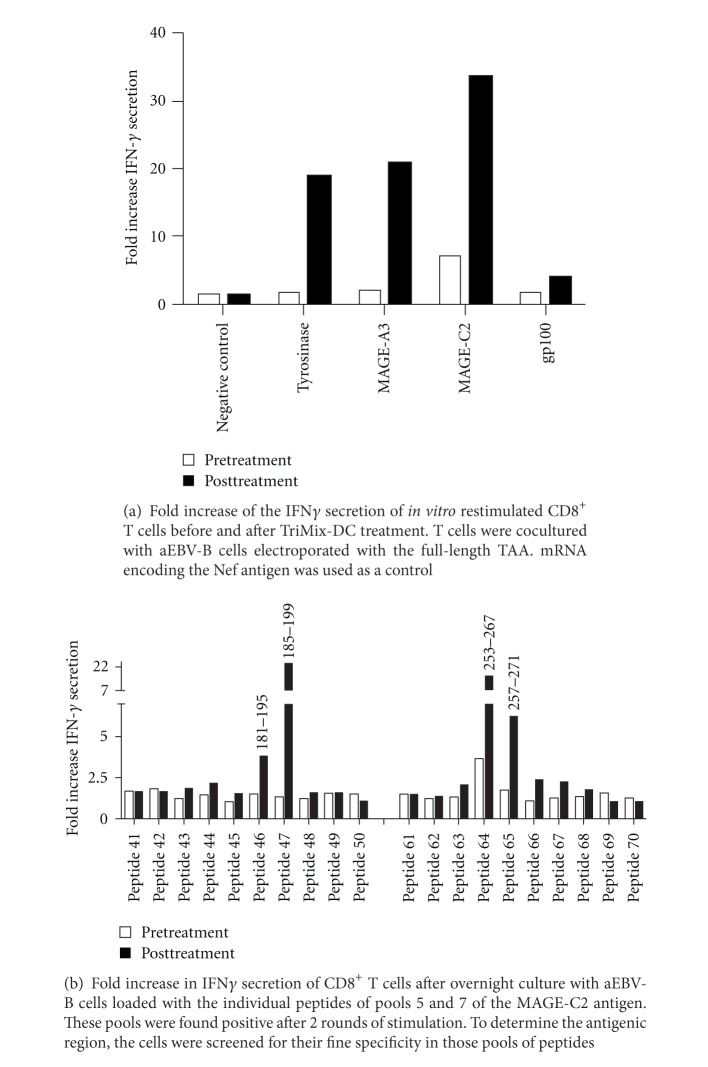
Treatment antigen-specific CD8^+^ T cells in peripheral blood.

An advantage of the immune monitoring of the peripheral blood is that preexisting immune responses can be investigated without the need for additional invasive procedures. In this group of patients, we found preexisting responses in 4 out of the 14 patients tested (29%) (Table 1). In all the patients with a detectable preexisting response, the response was further expanded after TriMixDC-MEL treatment, indicating that our DC therapy is able to expand responses *in vivo* (Figure 1(a)). 

Overall, when looking at the immune responses after DC therapy on the level of the tumor antigen, the CD8^+^ T-cell immune responses are very similar in the DTH skin biopsies and the peripheral blood of the patients. This comparison has not been performed by many groups. The group of Gaudernack [13, 14] defined a positive DTH immune reaction as induration and/or erythema after intradermal injection of the vaccine but did not investigate the antigen-specificity of the T cells in the DTH skin biopsies. This way, they documented 10/17 [13] and 3/10 [14] positive DTH reactions in their respective studies. On the other hand, antigen-specific T cells were documented more frequently in peripheral blood after antigen-specific restimulation: 7/17 [13] and 6/10 [14] immune responders were documented in their respective studies. The group of de Vries performed two studies where the antigen-specific T cells at both sites were investigated, and they detected more treatment antigen-specific T-cell responses in skin biopsies compared to the peripheral blood [15, 16]. In their first study [15], patients with colorectal cancer were vaccinated with CEA-peptide-loaded DCs. Seven out of 10 patients had CEA-specific SKILs detected by direct tetramer analysis, whereas none of the patients had antigen-specific T cells in the peripheral blood. In a more recent study [16], 27 patients with advanced melanoma were vaccinated ID/IV with a gp100 peptide. Although the majority of the vaccinated patients induced an induration at the DTH site, only 3 patients had antigen-specific SKILs detected by tetramer staining. No treatment antigen-specific tetramer positive CD8^+^ T cells could be detected in the blood. This was probably due to a too low frequency of antigen-specific T cells, since *in vitro* restimulation indicated that vaccination induced an increase in antigen-specific CD8^+^  T cells in the 3 patients with detectable antigen-specific T cells in DTH skin biopsies. Overall, these differences between groups may partly be explained by differences in the type of vaccine, the DC production, maturation, and antigen loading. Indeed, we have treated the patients with TriMixDC-MEL, which have a superior CD8^+^ T-cell stimulatory capacity *in vitro* [17]. Also the route of immunization might play a role [18–20]. In some cases, this difference could be explained by a difference of methods used for monitoring the peripheral blood responses: responses were more frequently observed after *in vitro* restimulation compared to direct *ex vivo* analysis, for example, by tetramer staining. Direct tetramer staining is only possible when the patient's HLA type is known and when investigating an immune response directed to a known and defined peptide. With our mRNA-based DC vaccine, the patient's HLA type is not known and the full-length TAA encoding mRNA ensures presentation of the full antigenic spectrum of epitopes, which impedes the use of direct tetramer staining. On the other hand, high-throughput tetramer-based methods have been developed. These tests could detect a large number of different T-cell epitopes requiring a limited amount of sample volume, without the need for *in vitro* stimulation [21–23]. Another method that can be used for the detection of antigen-specific responses without *in vitro* restimulation is ELISpot. However, in our hands, this resulted in a high level of aspecific responses. Unfortunately, the small number of patients investigated in our study does not allow any correlation between antigen-specific responses detected in DTH skin biopsies and peripheral blood and the clinical outcome.

When a positive immune response was observed in the SKILs or the peripheral CD8^+^ T cells of a patient, we attempted to characterize the antigenic region(s) recognized by the CD8^+^ T cells. To investigate this, cells were screened for their specificity after stimulation with aEBV-B cells pulsed with pools of 10 overlapping 15-mer peptides. We were able to investigate the antigenic regions recognized by the CD8^+^ T cells in both compartments of 4/10 patients mounting a CD8^+^ T-cell response in both compartments (Table 2, Figure 1(b)). As this screening method requires high amounts of both skin- and blood-derived CD8^+^ T cells, it was not possible to perform this screening for all patients included in this study. SKILs could not always be sufficiently expanded for detection of the fine specificity. Also for peripheral blood screening, leftover material from the vaccinal material was used and available material was a limiting factor in some cases. Nevertheless, in this limited group of patients, both regions containing previously described epitopes as well as regions that do not contain described epitopes were recognized [24]. These broad T-cell responses are a result of the use of full-length mRNA for TriMixDC-MEL treatment, resulting in the presentation of the complete antigenic repertoire without HLA restriction [1]. Some epitopes were recognized by CD8^+^ T cells isolated from both compartments, but the majority was either recognized by SKILs or peripheral T cells. 

Although only a small number of patients could be screened, these data suggest that there is a partial overlap between the antigenic regions recognized by CD8^+^ T cells situated in both compartments but that a certain degree of compartmentalization might take place. On the other hand, we cannot exclude that this phenomenon is due to technical limitations of our monitoring techniques. Both DTH skin biopsy and peripheral blood immune monitoring require *in vitro* culturing of the T cells and expansion of responding T lymphocytes up to detectable frequencies. As such, a bias towards T cells specific to certain peptides can arise, as some antigen-specific CD8^+^ T cells could be overgrown by others. 

In this study, we show that TAA-specific T cells can be detected in peripheral blood and DTH skin biopsies after TriMixDC-MEL treatment. Our results show that TAA-specific CD8^+^ T cells circulate in the blood stream. These T cells have the potential to access every place in the body via the circulation. This is of great importance for the eradication of metastasized disease. T cells that were found at the DTH skin biopsy highlight the capacity of the induced TAA-specific CD8^+^ T cells to leave the bloodstream and to migrate to tissues with enhanced expression of the treatment TAA. In addition, when tumor lesions are accessible, it should be interesting to also look at this site if antigen-specific T cells are present. The group of Tjin et al. [5] investigated the antigen specificity of tumor-infiltrating lymphocytes (TILs). They detected more antigen-specific TIL compared to antigen-specific T cells in the peripheral blood, indicating that T-cell responses could be missed when looking only at circulating T cells. It is thus of great importance to investigate the vaccinal antigen-specific immune response in as many compartments as possible to find all possible antigen-specific T lymphocytes. 

## 4. General Conclusion and Future Perspectives

We here report that treatment antigen-specific T cells can be detected both in the skin and the peripheral blood of melanoma patients. Although we see a high overlap between TAA-specific immune responses after TriMixDC-MEL treatment, the observed compartmentalization on the level of recognized antigenic regions highlights the importance of screening for immune responses in different compartments. We hypothesize that the detection of responses against treatment antigens in different body compartments might enable us to refine vaccine strategies and to optimize them. 

## Figures and Tables

**Table 1:** Monitoring the antigen-specific CD8^+^ T-cell responses in DTH skin biopsies and peripheral blood of melanoma patients participating in DC-therapy clinical trials. Positive responses were scored according to the strength of the immune response. Clinical outcome of the screened patients.

			DTH skin biopsy				Peripheral blood				Clinical outcome		
Patient code	Disease status	Injected DCs id/iv (×10^6^ cells)	gp100	Tyrosinase	MAGE-C2	MAGE-A3	gp100	Tyrosinase	MAGE-C2	MAGE-A3	BOR	PFS (m)	OS (m)
72	ED	65/0	—	—	—	—	—	—	—	—	SD	13.6	19.1
74	ED	43/0	—	+	+	—	—	—	++	—	SD	37.3	51.5+
75	ED	45/0	—	+	++	+	—	+	+++	++	SD	9.3	50.3+
76	NED	44/0	—	+	—	—	—	—	—	—	—	51.2+	51.2+
83	ED	40/0	—	++	+++	+++	—	—	+	+	PD	2.7	16.8
88	ED	54/0	+	++	++	+	—	+	+	+	PD	2.7	17.7
89	NED	16/0	—	+	+	+	—	+^∗^	++	—	—	45.6+	45.6+
92	ED	31/0	—	+	+	—	—	+	+	—	PD	3.4	17.2
98	ED	74/0	—	++	+++	++	—	+	+	+	PD	2.9	14.1
102	ED	20/4	+	+	+	+	—	+	++^∗^	+	CR	22.6+	22.6+
107	ED	4/20	—	+	—	+	—	+	+	+	PD	1.8	6.4
113	ED	4/20	+	+	+	—	+	++	++^∗^	+	PR	17.8+	17.8+
116	ED	4/20	—	—	—	—	+	+	+^∗^	—	SD	18.3+	18.3+
125	ED	0/24	—	—	—	—	—	—	—	—	CR	13.2+	13.2+

NED: no evaluable disease; ED: evaluable disease; BOR: best objective response; PFS: progression-free survival; m: months; OS: overall survival.

^∗^Indicates antigen-specific CD8^+^ T-cell responses that were present before the DC treatment.

—: no detectable response.

+: weak response (<10%).

++: moderate response (10–20%).

+++: strong response (>20%).

**Table 2:** Monitoring the fine specificity of the antigen-specific CD8^+^ T-cell responses in DTH skin biopsies and peripheral blood of melanoma patients.

Patient code	gp100		Tyrosinase		MAGE-C2		MAGE-A3	
	DTH skin biopsy	Peripheral blood	DTH skin biopsy	Peripheral blood	DTH skin biopsy	Peripheral blood	DTH skin biopsy	Peripheral blood
88			**87–** **95** ^a^	**87–** **95**				
				121–171				
				161–211				

89			121–171	385–403^∗^	281–331	375-371		

92					**41–91 **	**41–91 **		
					**161–211 **	**161–211 **		
					**241–291 **	**241–291**		
					281–331			
					321–371			

113	81–95	33–47	**201–215 **	**201–215 **	**285–299 **	165–179	65–79	77–91
	85–99	293–307	**205–219 **	**205–219 **	**289–303**	189–203	105–119	**165–179 **
			209–223	413–427		241–291^∗^	113–127	169–183
			285–299			257–271	161–175	297–311
			309–323			**285–299 **	**165–179 **	
			369–377			**289–303**	189–203	
			433–447				193–207	

^a^Numbers indicate the amino acid numbers of the antigenic region recognized by the CD8^+^ T cells.

^∗^Indicates antigen-specific CD8^+^ T-cell responses that were already present before the DC treatment.

Bold numbers were antigenic regions recognized by CD8^+^ T cells in both skin and peripheral blood.
